# Editorial: Cardiac Hypertrophy: From Compensation to Decompensation and Pharmacological Interventions

**DOI:** 10.3389/fphar.2021.665936

**Published:** 2021-04-26

**Authors:** Ya Liu, Xiongwen Chen, Hai-Gang Zhang

**Affiliations:** ^1^ Department of Pharmacology, Army Medical University (Third Military Medical University), Chongqing, China; ^2^ Cardiovascular Research Center, Temple University Lewis Katz School of Medicine, Philadelphia, PA, United States

**Keywords:** cardiac hypertrophy, cardiac fibrosis, heart failure, cardiac remodeling, heart function

**Editorial on the Research Topic** **Cardiac Hypertrophy: From Compensation to Decompensation and Pharmacological Interventions**

## Introduction

Cardiac hypertrophy is not a single disease, but a complication and a pathological process of many forms of cardiovascular disease, such as hypertension, congestive heart failure, valvular diseases and ischemic diseases, etc. (Yu et al., 2020). It is characterized by abnormal increases in mass and volume of myocardium at the whole heart level, in the size of individual myocyte at cell level, and in synthesis of protein and reprogramming expression of fetal genes at molecule level (Dodge-Kafka et al., 2019; Zhang et al., 2020). Myocardial hypertrophy is an important aspect of myocardial remodeling, which including not only the hypertrophic growth of myocytes but also the proliferation and activation of interstitial fibroblasts to produce intercellular matrix, namely fibrosis (Ge et al., 2021). These three pathological processes, cardiomyocyte hypertrophy, fibrosis and remodeling, are overlapped and closely related to each other.

Cardiac remodeling involves metabolic, mechanical, electrical, and structural alterations (Pitoulis and Terracciano, 2020). It is initially an adaptive response to pressure or volume overload. The myocardium undergoes hypertrophic growth as a compensatory measure aiming to improve myocardial contractility, reduce wall stress and maintain cardiac output. However, increase in mass and volume of myocardial tissue inevitably increase the oxygen consumption and dysfunction of energy metabolism in myocardial tissue (Nakamura and Sadoshima, 2018). Simultaneously, apoptosis, necrosis and autophagic cell death occur in cardiac myocytes, and proliferation and activation arise in fibroblasts, leading to interstitial fibrosis (Zhu and sun, 2018). The continued presence and evolving hypertrophy eventually lead to decompensation of heart function (Messerli et al., 2017; Oldfield et al., 2020). Many studies have demonstrated that ventricular hypertrophy and remodeling is associated with a significantly increased risk of heart failure, malignant arrhythmia, and even sudden death, and is thought to be an independent risk factor for increasing morbidity and mortality of cardiovascular diseases (He et al., 2020). Therefore, clinical guidelines in many countries and organizations have suggested it as primary goal to control or reverse cardiac hypertrophy in the therapeutics of hypertension and chronic heart failure (Di Palo and Barone, 2020).

Multiple signal transduction pathways involving Gq-phospholipase C-diacylglycerol (DAG)/inositol triphosphate (IP_3_), mitogen-activated protein kinases, calcineurin-nuclear factor of activated T cells (NFAT), phosphatidylinositol 3-kinase (PI3K)/protein kinase B (AKT), mammalian target of rapamycin (mTOR), transforming growth factor (TGF)-β, play important roles in the development of cardiac hypertrophy (Nakamura and Sadoshima, 2018), and complex crosstalks and feedbacks among them are present widely. Although the molecular mechanisms underlying cardiac hypertrophy have been extensively studied, there are still many uncharted territories that need to be explored thoroughly, especially the molecular mechanisms that control the transformation from compensation to decompensation.

During the transition from compensated hypertrophy to decompensation and deterioration of systolic heart function, apoptotic and necroptotic loss of cardiomyocytes, contractile dysfunction and massive fibrosis are key points. Heger et al. (2016) have documented in a review article that TGFβ superfamily play a central role. Some modulators of mitochondrial pores and transporters, such as NLR Family Pyrin Domain Containing 3 (NLRP3), adenine nucleotide translocator 1 (ANT1) and various miRNAs, etc., and regulators of adrenoceptor-mediated signaling pathway, such as SMAD4 and β-arrestin, are suggested to be switch molecules for this transition. Zhen et al. (2021) recently reported that signal transducers and transcriptional activation 1 (STAT1) was able to enhance mitochondrial function and prolong the time of compensation period through uncoupling protein 2 (Ucp2)/dynamin-related protein 1 (Drp1) signaling pathway.

In recent years, noncoding RNAs including microRNAs (miRNAs), circular RNAs (circRNAs) and long non-coding RNAs (lncRNAs) have gained more and more attention in the cardiac hypertrophy research field (Heger et al., 2016; He et al., 2020). Multiple miRNAs, such as miR-1, miR-21, miR-132/212, have been uncovered to regulate the process of cardiac hypertrophy *via* influencing the expression of a number of target genes including β1-adrennergic receptor, TGFβ1 receptor III, matrix metalloproteinase-2, connexin 43 (Wang et al., 2016; Mushtaq et al., 2020). Meanwhile, various lncRNAs have been shown to play important roles in both cardiac development and pathological cardiac remodeling. Targeting noncoding RNAs seems to open up a novel strategy for the treatment of cardiac hypertrophy.

This Research Topic aims to provide a platform for discussing about the molecular mechanisms of cardiac hypertrophy and remodeling, as well as the potential drug targets. Nine articles from 5 countries are collected here. Huang et al. reported that cellular communication network factor 5 (CCN5) could inhibit fibroblast-to-myofibroblast transition and suggested it might serve as a potential biomarker for estimating cardiac fibrosis in hypertensive patients as well as a novel therapeutic target. Stafford et al. demonstrated the role of IL-10 signal in the development of cardiac hypertrophy and indicated it might become a therapeutic target. Xiang et al. contributed an interesting review paper focused on the variation of energy metabolism in exercise-induced physiological myocardial hypertrophy, which may antagonize the progress of pathological hypertrophy.

Transfer RNA derived small RNAs (tsRNAs) have recently emerged as important modulators of protein translation and shown to possess varied functions. Cao et al. reviewed that nuclear and mitochondrial tsRNAs, including tRNA halves (tiRNAs), and tRNA fragments (tRFs), exert crucial effects in the development of pathological and decompensated cardiac hypertrophy, implying new therapeutic targets to battle cardiovascular disease.

Thanks to the advantages of multi-target effects, traditional Chinese medicine has attracted growing interests in the treatment of cardiovascular diseases. Yan et al. demonstrated gallic acid could effectively attenuate angiotensin II-induced hypertension and vascular dysfunction through inhibit immunoproteasome. In the article by Li et al., it was found that triptolide, the major active component of the Chinese medicinal herb *Tripterygium wilfordii* Hook F, improved cardiac hypertrophy *via* correction of the unbalanced expression of various cell cycle regulators.

Remarkably, cardiac hypertrophy is not only a complication (result) of multiple cardiovascular diseases, but also a pathological process (reason) of them. Due to the complexity of signaling pathway networks underlying cardiac hypertrophy, it might be difficult to block and reverse cardiac hypertrophy by targeting a single molecule. Therefore, further research needs to be performed, and interventions targeting immune-inflammatory reaction and transcription factors may bring the promise to treat cardiac hypertrophy.
